# Differences in Phenotypic Plasticity between Invasive and Native Plants Responding to Three Environmental Factors

**DOI:** 10.3390/life12121970

**Published:** 2022-11-25

**Authors:** Luna Zhang, Anqun Chen, Yanjiao Li, Duohui Li, Shiping Cheng, Liping Cheng, Yinzhan Liu

**Affiliations:** 1 International Joint Research Laboratory for Global Change Ecology, Laboratory of Biodiversity Conservation and Ecological Restoration, School of Life Sciences, Henan University, Kaifeng 475004, China; 2 Henan Key Laboratory of Germplasm Innovation and Utilization of Eco-Economic Woody Plant, Key Laboratory for Value Realization of Ecological Products of Mountains-Rivers-Forests-Farmlands-Lakes-Grasslands in Pingdingshan City, Pingdingshan University, Pingdingshan 467000, China

**Keywords:** plant invasion, phenotypic plasticity, resource constraints, functional traits, nutrients, water, light

## Abstract

The phenotypic plasticity hypothesis suggests that exotic plants may have greater phenotypic plasticity than native plants. However, whether phenotypic changes vary according to different environmental factors has not been well studied. We conducted a multi-species greenhouse experiment to study the responses of six different phenotypic traits, namely height, leaf number, specific leaf area, total biomass, root mass fraction, and leaf mass fraction, of native and invasive species to nutrients, water, and light. Each treatment was divided into two levels: high and low. In the nutrient addition experiment, only the leaf mass fraction and root mass fraction of the plants supported the phenotypic plasticity hypothesis. Then, none of the six traits supported the phenotypic plasticity hypothesis in the water or light treatment experiments. The results show that, for different environmental factors and phenotypes, the phenotypic plasticity hypothesis of plant invasion is inconsistent. When using the phenotypic plasticity hypothesis to explain plant invasion, variations in environmental factors and phenotypes should be considered.

## 1. Introduction

Plant invasion is one of the most severe threats to terrestrial ecosystems globally [1,2] and is thus one of the most pressing environmental problems worldwide in the 21st century [3]. Due to climate change and human activities, the frequency and extent of plant invasions are increasing. Plant invasions have considerable impacts on the structure and function of terrestrial ecosystems [4,5,6] and drastically threaten economic development [7,8] and human health [9,10]. Accordingly, efforts to determine the mechanisms of spread and control of invasive plants are increasing. Identifying factors associated with the spread of invasive plants is becoming a key topic of research in invasion ecology. Previous studies have ascribed successful invasion to domination in nutrient resource competition [11], allelopathy [12], and the enemy release hypothesis [13]. In addition, some invasion biologists have suggested that phenotypic plasticity plays an important role in plant invasion [14,15,16,17,18,19,20,21].

Phenotypic plasticity is the ability of plants to produce different phenotypes under different environmental conditions [14,15], which is one of the ways in which organisms adapt to environmental changes. Phenotypic plasticity is a key mechanism for successful invasion by invasive plants [16,17,18]. After colonizing in a new environment, invasive plants may exhibit a plastic response to novel selection pressures, enhancing their local fitness and advantages in competition with local species, which enables successful invasion [19]. Phenotypic plasticity is positively correlated with the invasive ability of invasive species in many studies [20,21,22,23]. In a common garden experiment, invasive species exhibited greater plasticity than native plants [22]. Higher plasticity was found in the invasive species *Sphagneticola trilobata* than in a local congener, *Sphagneticola calendulacea,* which promotes its invasion [24,25]. However, some studies did not support the hypothesis that invaders have higher phenotypic plasticity. In another common garden experiment, the phenotypic plasticity of invasive plants was lower than that of native plants [26]. For example, the results of a mesocosm pot experiment showed that although increasing water availability increased plant biomass, the magnitude of the effects did not differ between native and invasive species [27]. This may be because plants respond to different environmental factors with different traits. Plant growth is affected by many environmental factors, including nutrient content [28,29], soil water availability [28,30,31], and light intensity [29,32,33]. However, certain traits in certain species are plastic in response to specific environmental changes. It is likely that measuring plastic traits is key in identifying consistently higher plasticity in invasive species than in native species.

We performed a pot experiment to test how three environmental factors with two levels affected three native species and three invasive species. We measured six typical phenotypic indices, namely height, specific leaf area (SLA), biomass, leaf number, root biomass fraction, and leaf biomass fraction. The objectives of this study were to: (1) test whether the response patterns of different traits of native and invasive species are different for each environmental factor, and (2) determine whether these traits are more sensitive to specific environmental factors. We hypothesized that the phenotypic plasticity variances between invasive and native plants are environment- and trait-dependent.

## 2. Materials and Methods

### 2.1. Study Species

We chose three invasive species and three native species as the subjects of the study (Table 1). These native and invasive species were selected from the invasive alien species of China (IASC) and Ma et al. (2013) [34]. These six species included three pairs of congeneric species, each comprising one native species and one invasive species.

### 2.2. Experimental Set-Up

**Figure 1:**
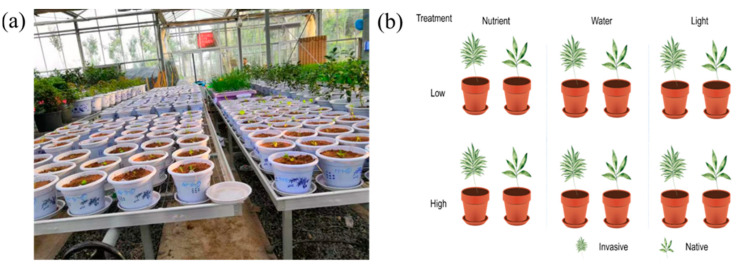
Greenhouse experiment set-up (**a**) and schematic representation (**b**) of the experimental design.

To compare differences in phenotypic plasticity between invasive and native plants under different environmental conditions, we conducted pot experiments in a greenhouse with an area of approximately 400 m^2^ at Pingdingshan University (Figure 1) with the six selected species. We initially performed seedling culturing, as described by Liu et al. (2021) [35]. We then applied an initial dose of 5 g NPK slow-release fertilizer (Osmocote) per pot to the substrate in all pots for the water and light experiments. Based on previous experiments [35], some species germinated later than others, so we seeded these species on different dates to ensure that the seedlings were at a similar stage of development (i.e., similar size and height) at the start of the experiment.

There were three treatments in our experiment that did not interact with each other, namely nutrients, light, and water. Each treatment had two levels (high and low), and each species had eight replicates for each factor level. We filled 288 circular 2 L plastic pots for six treatments (low nutrients, high nutrients, low water, high water, shading, and ambient light × 6 species × 8 replicates) with a 1:1 mixture of sand and fine vermiculite. We transplanted each seedling into the center of a pot and watered it until the medium was saturated. To avoid nutrient leakage from the pot after watering, we placed a plastic plate under each pot. The pots were separated into three groups to test the effects of water, nutrients, and light on plant functional traits (Figure 1). 

For the light and water treatments, 5 g of NPK slow-release fertilizer was added to each pot of substrate during planting. For the high-nutrient treatment, 6 g of slow-release fertilizer was added to each pot, and 1 g was added to the low-nutrient treatment pots. For the high-water treatment, 300 mL of water was added to each pot every three days, and 100 mL of water was added to each pot every three days for the low-water treatment. A single layer of black shading net was used to create shade for the shading treatment; the light intensity of the shading treatment was reduced by 30% compared with natural light. Under the ambient light treatment, no measures were added; the potted plants received natural sunlight.

### 2.3. Trait Measurements and Phenotypic Plasticity Index

We measured the initial height of each plant with a ruler on the second day after transplantation and measured all traits eight weeks after initiation of the experimental treatment. We measured the height of each plant with a ruler, counted the number of leaves, and then collected the leaves at the midpoint according to the height of each plant to measure the leaf area using a scanner before measuring the leaf biomass [36]. Next, we carefully washed away the remaining substrate from the plant roots. The aboveground and belowground parts of each plant were oven-dried at 75 °C for 48 h to a constant weight, and the biomass was then weighed. We calculated the root mass fraction (RMF) from the ratio of root biomass to total biomass and calculated the leaf mass fraction (LMF) from the ratio of leaf biomass to total biomass. We calculated the plasticity index to estimate the phenotypic plasticity of the six traits, following Valladares et al. [37] and Gruntman et al. [33].

### 2.4. Statistical Analysis

All statistical analyses were conducted using R version 4.0.3. We fit linear mixed-effects models using the LME function of the “NLME” package [38] to analyze the differences in phenotypic plasticity between native and invasive plants under different environmental conditions. We defined initial height, leaf number, SLA, total biomass, RMF, and LMF as the response variables, and we defined water and plant origin (invasive or native species), nutrients and plant origin, and light and plant origin as fixed effects in the models for all three treatments. Because the initial state of the plants may have contributed to differences in the final data results, we added initial height as a covariable in the model. To account for the non-independence of individuals of the same plant species and for phylogenetic non-independence of related species, we included the identity of the target species and its corresponding family as random factors in all the models. We also tested the response of different variables to the environmental changes. In general, the response of the species was consistent across the groups. To meet the assumption of normality, we checked the distribution of the data and transformed it if the data did not fit the normal distribution. When analyzing the nutrient treatment results, plant height and SLA were *ln*-transformed, total biomass was square-root transformed, and RMF and LMF were cube-root transformed. When analyzing the water treatment results, the height, SLA, RMF, and LMF were *ln*-transformed. When analyzing the light treatment results, the SLA, total biomass, and RMF were *ln*-transformed.

## 3. Results

### 3.1. Trait Responses to Nutrient Treatment

**Figure 2:**
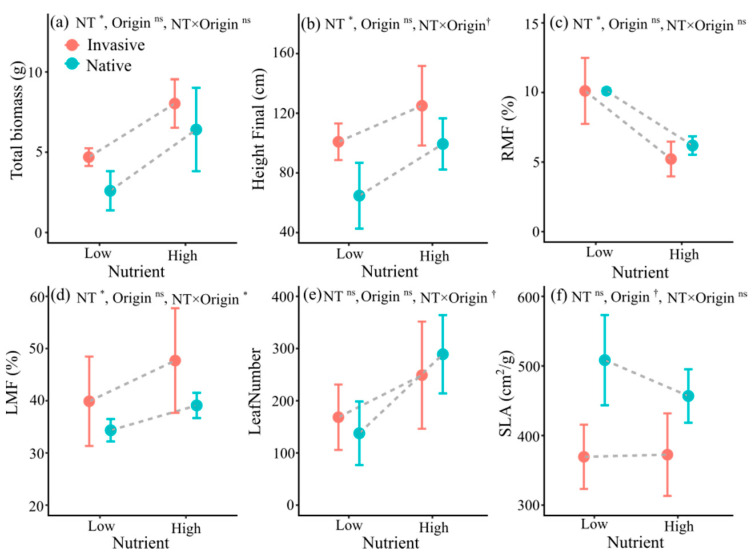
Mean values (±SE) of total biomass (**a**), height (**b**), RMF (**c**), LMF (**d**), leaf number (**e**), and SLA (**f**) of invasive and native plants under different nutrient levels. Error bars represent standard errors, *n* = 8 replicates per treatment. ^†^ *p* < 0.1; * *p* < 0.05; ns, non-significant; other abbreviations as in Table 2.

Plants receiving high-nutrient treatment had increased total biomass and height (Table 2, Figure 2). The effects of nutrients on total biomass and height did not differ with plant origin (Table 2). High-nutrient treatment significantly decreased the RMF (Table 2, Figure 2c) but significantly elevated the LMF (Table 2, Figure 2d). The effect of nutrients on the LMF varied significantly with plant origin. High nutrient levels increased the LMF of the invasive plants by 19.56% but only increased the LMF of the native plants by 13.86% in the experiment (Figure 2d). High-nutrient treatment did not affect the leaf number (Table 2, Figure 2e) or the SLA (Table 2, Figure 2f).

### 3.2. Trait Responses to Water Treatment

**Figure 3:**
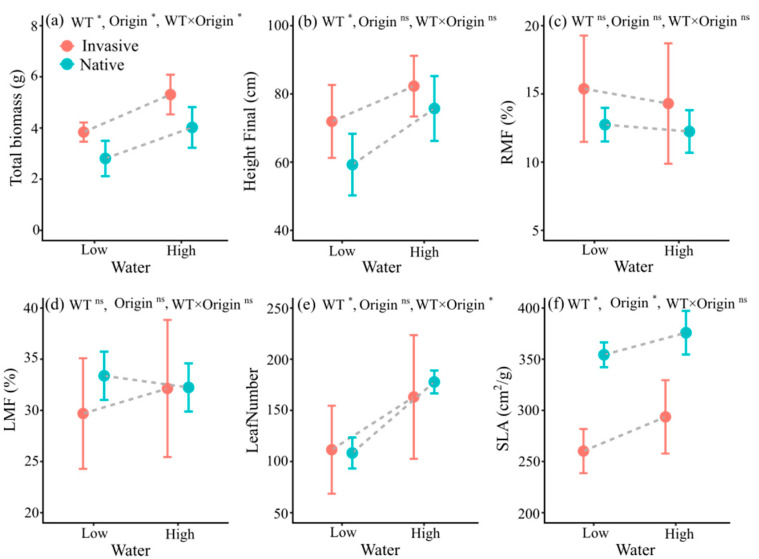
Mean values (± SE) of total biomass (**a**), final height (**b**), RMF (**c**), LMF (**d**), leaf number (**e**), and SLA (**f**) of invasive and native plants under different water levels. Error bars represent standard errors, *n* = 8 replicates per treatment. * *p* < 0.05; ns, non-significant; other abbreviations as in Table 2.

The responses of the total biomass to water varied significantly with plant origin (Table 2). High water content increased the total biomass of invasive and native plants by 38.22% and 43.15%, respectively (Table 2, Figure 3a). In addition, high water content significantly increased plant height (Table 2, Figure 3b). The effect of water on plant height did not differ with plant origin (Table 2). High-water treatment did not affect RMF (Table 2, Figure 3c) or LMF (Table 2, Figure 3d). The effect of water on leaf number varied significantly with plant origin (Table 2), where the high-water treatment increased the leaf number in invasive and native plants by 46.21% and 64.08%, respectively (Figure 3e). High water significantly increased SLA, but the magnitude of this effect did not differ according to plant origin (Table 2, Figure 3f).

### 3.3. Trait Responses to Light Treatment

**Figure 4:**
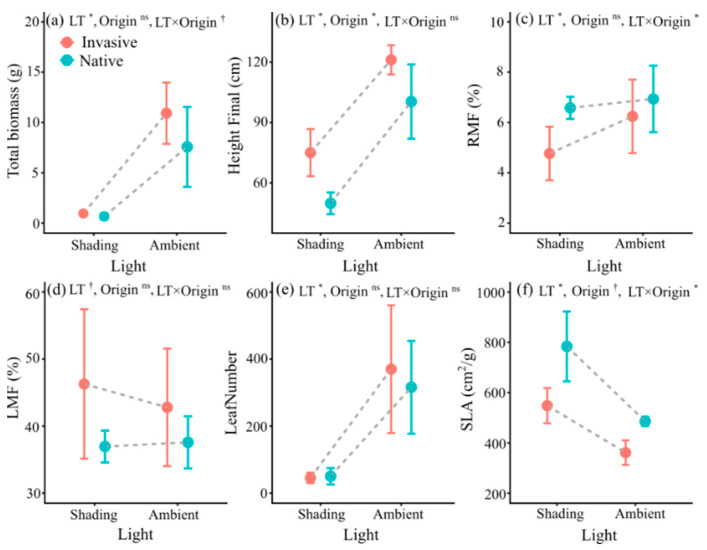
Mean values (± SE) of total biomass (**a**), final height (**b**), RMF (**c**), LMF (**d**), leaf number (**e**), and SLA (**f**) of invasive and native plants under different light levels. Error bars represent standard errors, *n* = 8 replicates per treatment. ^†^ *p* < 0.1; * *p* < 0.05; ns, non-significant; other abbreviations as Table 2.

Total biomass (Table 2, Figure 4a) and height (Table 2, Figure 4b) under shaded conditions were significantly lower than those under ambient light. The responses of total biomass and height to light did not vary with plant origin (Table 2). Yet, the effects of light on the RMF varied significantly with plant origin (Table 2, Figure 4c). Shading decreased the RMF of invasive plants by 1.48% and that of native plants by 0.36% (Table 2, Figure 4c). Shading did not affect the LMF (Figure 4d). Shading significantly decreased the leaf number (Table 2, Figure 4e). However, the effect of light on the leaf number did not vary with plant origin (Table 2). Shading significantly elevated the SLA, and the effect differed significantly with plant origin (Table 2, Figure 4f). Shading significantly increased the SLA of the invasive and native plants by 51.71% and 61.34%, respectively (Figure 4f).

### 3.4. Variation of Phenotypic Plasticity Index

The phenotypic plasticity index varied according to species, plant traits, and environmental factors (Table S2). The phenotypic plasticity index for the root mass fraction and leaf mass fraction was significantly higher for invasive plants than for native plants under the nutrient treatment (Table 3). However, the phenotypic plasticity of invasive plants was not higher than that of native species under water or light treatment (Table 3).

## 4. Discussion

### 4.1. Phenotypic Plasticity of Invasive and Native Plants under Different Nutrient Levels

With increased nutrient availability, plants produce more biomass but decrease their root mass fraction. This is in line with the results of previous research [39,40,41]. The most basic response of plants to environmental change is to change the biomass allocation among their organs. Given that roots are predominantly responsible for nutrient absorption [40,42,43], plants often allocate more biomass to the roots to ensure their survival when insufficient nutrients are available than in normal conditions [44]. For example, in an experiment with 29 plant species, the root biomass fraction was found to be significantly lower when plants were grown under high-nutrient conditions than under low-nutrient conditions [28]. This is consistent with the optimal partitioning theory, which states that plants should allocate biomass to structures according to the degree of limitation of resources [45,46].

In the absence of nutrient limitations, native plants often allocate additional resources to aboveground parts to improve their competitiveness, meaning the height advantage of invasive plants is reduced. Moreover, with nutrient addition, the competitive advantage of invasive plants may not be reflected in the biomass since nutrients were already naturally abundant. In this regard, our findings are inconsistent with those of most previous studies [23,40]. It has been demonatrated that the biomass of some native species may be more sensitive to nutrient enrichment than that of invasive species [27]. However, our results showed no significant difference in biomass between invasive and native species following nutrient addition. Therefore, biomass plasticity may not be the most relevant mechanism for explaining invasion along the gradient of nutrient limitation in our experiment.

Our results also showed that the SLA and leaf number did not respond strongly to nutrient addition. This is likely because plant leaves are organs of transpiration and photosynthesis [47] and may not be sensitive to changing nutrient conditions. However, the leaf mass fraction of invasive plants gradually revealed an advantage held by invasive species with increasing nutrient availability. The leaf mass fraction correlates with increasing nutrients [25,40,48], and we saw that the leaf mass fraction of invasive plants increased faster than that of native plants. Plants increase their leaf mass fraction to obtain more light when nutrients are not limited [49], and this effect was weaker in native plants than in invasive plants. The leaf mass fraction results were consistent with the plasticity hypothesis in the fertilization experiment.

### 4.2. Phenotypic Plasticity of Invasive and Native Plants under Different Water Levels

The plant height, leaf number, specific leaf area, and total biomass of the invasive and native plants increased, but biomass allocation did not change under the high-water treatment. Biomass is likely the most feasible variable for quantifying plant resource allocation across plant functional traits [50,51]. Plants alter their normal morphological and physiological processes under water stress [40]. A meta-analysis showed that drought significantly decreases the aboveground biomass of plants [52], predominantly because roots are the main organs that absorb water. With relatively well-developed root systems, plants can absorb good amounts of water from the soil for survival. However, biomass allocation did not change with high-water treatment, which is inconsistent with the findings of most previous studies [40,52]. This indicates that the response of biomass allocation to water may vary according to plant species. When water availability decreased in our experiment, plant growth was negatively impacted. This may be because leaves consume considerable amounts of water [30]. Therefore, when water is limited, the number of leaves is likely to be reduced, which reduces water loss.

Invasive plant species generally have more biomass than native species, particularly under adequate water conditions [53]. When the amount of favorable resources increases, invasive plants may be able to obtain resources more rapidly than native plants. Therefore, resource availability plays an important role in colonization by invasive plants [54]. Our results are consistent with those of previous studies. Goergen and Daehler (2001) found that native plants are less sensitive to drought than their invasive counterparts [55]. A potential reason for this is that invasive species may be more plastic than noninvasive species [16,55,56]. This would mean that invasive species have competitive advantages under conditions with high water availability.

Native and invasive plants are negatively affected by water loss, and invasives are more strongly affected than natives. This supports the theory that extreme water scarcity may hinder invasion because invasive species respond more strongly to favorable and adverse climatic conditions than native species [57]. However, our results showed that the effects of water treatment on the number of leaves varied with plant origin. When water availability was limited, the reduction in the number of leaves of invasive plants was lower than that of native species, indicating that invasive plants were more responsive to water restriction than native plants. However, the consistent phenotypic plasticity index observed between the invasive and native plants indicates that the leaf number did not follow the phenotypic plasticity hypothesis in our experiment. This finding may be explained by interspecific variation. Studies with greater numbers of invasive and native plants are needed to confirm whether the phenotypic plasticity hypothesis is applicable in the context of changing water levels.

### 4.3. Phenotypic Plasticity of Invasive and Native Plants under Different Light Levels

Total biomass, plant height, leaf number, and root biomass fraction decreased in the shade treatment, while the specific leaf area and leaf number increased. Light is the main limiting factor for plant growth, and a decrease in light intensity usually inhibits plant growth, resulting in stunted growth [58,59,60]. With insufficient light, plants usually produce large, thin leaves to improve their ability to obtain light [61] since the specific leaf area is an important functional trait that may affect light interception, along with the leaf lifespan [62]. It has been found that the response of the specific leaf area to shading is often highly plastic [63].

In this research, the specific leaf area of native species increased more than that of invasive species under shading. This is inconsistent with a previous study that found that the response of a specific species to shade was unrelated to specific leaf area plasticity [64]. However, another study showed that invasive species had a lower specific leaf area when shaded [33]. This difference in plasticity suggests that invasive species may be pre-adapted to succeed in novel environments. In one study, artificial shading was used to investigate the phenotypic plasticity of invasive plants in response to different light intensities. Compared to the control, shading significantly increased the ratio of leaf area, specific leaf area, and leaf biomass to total biomass [65]. However, under shading treatment, the root mass fraction of invasive plants decreased more than that of native plants. As such, the root mass fraction of invasive plants was found to be more plastic and sensitive to light changes than that of native plants. Yet, the plasticity of the specific leaf area of native plants was stronger than that of invasive species after shading. Meanwhile, invasive plants were more plastic than native species in terms of leaf area and became more invasive with increasing light intensity.

## 5. Conclusions

We have shown that although nutrients, water, and light altered most of the phenotypic traits of invasive and native plants, only the leaf mass fraction and root mass fraction of invasive plants were more plastic than those of native plants with elevated nutrient availability. Meanwhile, the phenotypic plasticity index of invasive plants was not higher than that of native species in the water or light treatment. Although other studies have shown different results, they often studied different variables and treatment levels, and they rarely compared the applicability of the phenotypic plasticity hypothesis for the same groups of native and invasive species under different environmental factors. Our results demonstrate that the phenotypic plasticity of invasive and native species is environment- and trait-dependent. We also found that different plant characteristics contribute to plants’ success in obtaining various resources. Therefore, when using phenotypic plasticity to explain the invasion success of invasive plants, the right traits for local environmental changes should be chosen. This may explain why previous studies have shown mixed results regarding the role of phenotypic plasticity in plant invasion success. 

We acknowledge that our findings need to be confirmed through additional studies. We did not consider interactions among different environmental factors in this experiment, which can be highly important in ecological studies. Further experiments are needed to verify whether different environmental factors interact to influence phenotypic plasticity. The choice of experimental subjects is also important since phenotypic plasticity may vary among different combinations of plant species. In our experiments, we only considered vegetative plant traits. However, the generative phase of plant development and reproductive strategies are also important. Further research encompassing these is thus needed to confirm the applicability of the phenotypic plasticity hypothesis.

## Figures and Tables

**Table 1:** Plant species used in the experiment.

Species	Family	Origin of the Plants
*Sphagneticola trilobata*	Asteraceae	Invasive
*Alternanthera philoxeroides*	Amaranthaceae	Invasive
*Hydrocotyle vulgaris*	Araliaceae	Invasive
*Sphagneticola calendulacea*	Asteraceae	Native
*Alternanthera sessilis*	Amaranthaceae	Native
*Hydrocotyle sibthorpioides*	Araliaceae	Native

**Table 2:** Results from linear mixed-effects models (χ^2^ value) examining the effects of nutrient (NT), water (WT), light (LT), and interactions between origin (invasive vs. native) with NT, WT, or LT on height, leaf number, specific leaf area (SLA), total biomass, root mass fraction (RMF), and leaf mass fraction (LMF) of the plants. * *p* < 0.05; ** *p* < 0.01; *** *p* < 0.001.

Fixed Effects	Height	Leaf Number	SLA	Total Biomass	RMF	LMF
**Nutrient**						
Initial Height	7.46 **	6.38 *	0.11	17.94 ***	15.17 ***	3.29
NT	6.59 **	1.69	0.01	22.86 ***	39.88 ***	4.50 *
Origin	2.27	0.48	2.77	1.66	0.43	0.84
NT × Origin	3.55	3.32	0.18	0.03	1.01	11.50 ***
Random Effects	**SD**	**SD**	**SD**	**SD**	**SD**	**SD**
Genus	0.325	8.531	1.585	0.251	0.156	4.443
Species	0.333	1.116	0.210	0.655	0.136	0.298
R^2^						
Marginal	0.182	0.119	0.288	0.238	0.267	0.151
Conditional	0.540	0.583	0.740	0.521	0.496	0.753
**Water**						
Initial Height	0.01	0.06	3.85 *	4.17 *	5.60 *	12.49 ***
WT	22.55 ***	227.17 ***	12.78 ***	43.83 ***	1.41	0.92
Origin	1.77	0.65	5.48 *	6.13 *	0.30	0.06
WT × Origin	0.36	10.64 ***	0.15	4.52 *	0.35	0.03
Random Effects	**SD**	**SD**	**SD**	**SD**	**SD**	**SD**
Genus	0.201	0.296	1.084	0.980	0.227	0.135
Species	0.112	0.495	0.132	0.273	0.274	0.185
R^2^						
Marginal	0.173	0.129	0.551	0.451	0.071	0.095
Conditional	0.555	0.734	0.819	0.788	0.908	0.601
**Light**						
Initial Height	0.88	1.16	0.65	5.49 *	0.01	0.83
LT	5.78 *	85.57 ***	91.60 ***	153.32 ***	3.85 *	2.94
Origin	4.09 *	0.32	3.42	1.42	1.27	0.78
LT × Origin	0.05	0.01	4.67 *	3.18	3.86 *	0.09
Random Effects	**SD**	**SD**	**SD**	**SD**	**SD**	**SD**
Genus	0.004	0.736	1.273	0.001	0.251	3.392
Species	14.760	0.126	0.197	1.062	0.193	11.054
R^2^						
Marginal	0.603	0.573	0.558	0.574	0.092	0.112
Conditional	0.781	0.872	0.817	0.806	0.390	0.891

**Table 3:** Mean values (± SE) of phenotypic plasticity index for final height, specific leaf area (SLA), leaf number, total biomass, root mass fraction (RMF), and leaf mass fraction (LMF) of native and invasive species under nutrient, light, and water treatments. Numbers following “±” represent standard errors, *n* = 3 replicates per plant type. Different letters after the number represent significance at *p* < 0.05.

Treatment	Variables	Invasive	Native
Nutrient	Final Height	0.189 ± 0.14a	0.372 ± 0.12a
	Leaf Number	0.314 ± 0.12a	0.597 ± 0.15a
	SLA	0.049 ± 0.02b	0.148 ± 0.09a
	Total Biomass	0.364 ± 0.16a	0.674 ± 0.10a
	RMF	0.485 ± 0.01a	0.387 ± 0.06b
	LMF	0.165 ± 0.01a	0.109 ± 0.10b
Water	Final Height	0.132 ± 0.05a	0.222 ± 0.02a
	Leaf Number	0.334 ± 0.04a	0.396 ± 0.05a
	SLA	0.105 ± 0.03a	0.072 ± 0.03a
	Total Biomass	0.264 ± 0.05a	0.312 ± 0.06a
	RMF	0.098 ± 0.05b	0.172 ± 0.01a
	LMF	0.074 ± 0.04a	0.077 ± 0.04a
Light	Final Height	0.369 ± 0.13a	0.480 ± 0.08a
	Leaf Number	0.795 ± 0.10a	0.826 ± 0.03a
	SLA	0.342 ± 0.01a	0.351 ± 0.08a
	Total Biomass	0.900 ± 0.04a	0.876 ± 0.04a
	RMF	0.233 ± 0.05a	0.252 ± 0.07a
	LMF	0.061 ± 0.03a	0.172 ± 0.08a

## Data Availability

All data for this article can be found at https://doi.org/10.5061/dryad.mcvdnck4f (accessed on 25 October 2022).

## Supplementary Materials

The following supporting information can be downloaded at: https://www.mdpi.com/article/10.3390/life12121970/s1, Table S1. Mean (± SE) values of height final, specific leaf area (SLA), leaf number, total biomass, root mass fraction (RMF) and leaf mass fraction (LMF) at the end of experiment of native and invasive species growing under nutrient, light and water treatments. Table S2. Phenotypic plasticity index of height final, specific leaf area (SLA), leaf number, total biomass, root mass fraction (RMF) and leaf mass fraction (LMF) of six species under nutrient, light and water treatments.

## References

[1] Vitousek P.M., D’Antonio C.M., Loope L., Westbrooks R. (1996). Biological invasions as global environmental change. Am. Sci..

[2] Mack R.N., Simberloff D., Lonsdale W.M., Evans H., Clout M., Bazzaz F.A. (2000). Biotic invasions: Causes, epidemiology, global consequences, and control. Ecol. Appl..

[3] Christian C.E. (2001). Consequences of a biological invasion reveal the importance of mutualism for plant communities. Nature.

[4] Yang W., Zhao H., Leng X., Cheng X., An S. (2017). Soil organic carbon and nitrogen dynamics following Spartina alterniflora invasion in a coastal wetland of eastern China. Catena.

[5] Prass M., Ramula S., Jauni M., Setälä H., Kotze D.J. (2021). The invasive herb Lupinus polyphyllus can reduce plant species richness independently of local invasion age. Biol. Invasions.

[6] Ni G., Zhao P., Huang Q., Zhu L., Hou Y., Yu Y., Ye Y., Ouyang L. (2020). Mikania micrantha invasion enhances the carbon (C) transfer from plant to soil and mediates the soil C utilization through altering microbial community. Sci. Total Environ..

[7] Early R., Bradley B.A., Dukes J.S., Lawler J.J., Olden J.D., Blumenthal D.M., Tatem A.J. (2016). Global threats from invasive alien species in the twenty-first century and national response capacities. Nat. Commun..

[8] Fantle-Lepczyk J.E., Haubrock P.J., Kramer A.M., Cuthbert R.N., Turbelin A.J., Crystal-Ornelas R., Courchamp F. (2022). Economic costs of biological invasions in the United States. Sci. Total Environ..

[9] Vilà M., Espinar J.L., Hejda M., Hulme P.E., Jarosik V., Maron J.L., Pergl J., Schaffner U., Sun Y., Pysek P. (2011). Ecological impacts of invasive alien plants: A meta-analysis of their effects on species, communities and ecosystems. Ecol. Lett..

[10] Courchamp F., Fournier A., Bellard C., Bertelsmeier C., Bonnaud E., Jeschke J.M., Russell J.C. (2017). Invasion biology: Specific problems and possible solutions. Trends Ecol. Evol..

[11] Shea K., Chesson P. (2002). Community ecology theory as a framework for biological invasions. Trends Ecol. Evol..

[12] Callaway R.M., Ridenour W.M. (2004). Novel weapons: Invasive success and the evolution of increased competitive ability. Front. Ecol. Environ..

[13] Maron J.L., Vila M. (2001). When do herbivores affect plant invasion? Evidence for the natural enemies and biotic resistance hypotheses. Oikos.

[14] Bradshaw A.D. (1965). Evolutionary significance of phenotypic plasticity in plants. Adv. Genet..

[15] Pigliucci M., Murren C.J., Schlichting C.D. (2006). Phenotypic plasticity and evolution by genetic assimilation. J. Exp. Biol..

[16] Richards C.L., Bossdorf O., Muth N.Z., Gurevitch J., Pigliucci M. (2006). Jack of all trades, master of some? On the role of phenotypic plasticity in plant invasions. Ecol. Lett..

[17] Zenni R.D., Lamy J.B., Lamarque L.J., Porté A.J. (2014). Adaptive evolution and phenotypic plasticity during naturalization and spread of invasive species: Implications for tree invasion biology. Biol. Invasions.

[18] Zheng Y., Burns J.H., Wang R., Yang A., Feng Y. (2021). Identity recognition and the invasion of exotic plant. Flora.

[19] Wang M., Ruan W., Kostenko O., Carvalho S., Hannula S.E., Mulder P.P., Bezemer T.M. (2019). Removal of soil biota alters soil feedback effects on plant growth and defense chemistry. New. Phytol..

[20] Davidson A.M., Jennions M., Nicotra A.B. (2011). Do invasive species show higher phenotypic plasticity than native species and if so, is it adaptive? A meta-analysis. Ecol. Lett..

[21] Kaur A., Kaur S., Singh H.P., Batish D.R., Kohli R.K. (2019). Phenotypic variations alter the ecological impact of invasive alien species: Lessons from Parthenium hysterophorus. J. Environ. Manage..

[22] Hiatt D., Flory S.L. (2020). Populations of a widespread invader and co-occurring native species vary in phenotypic plasticity. New. Phytol..

[23] Kleine S., Weissinger L., Müller C. (2017). Impact of drought on plant populations of native and invasive origins. Oecologia.

[24] Sun Z., Chen Y., Schaefer V., Liang H., Li W., Huang S., Peng C. (2015). Responses of the hybrid between *Sphagneticola trilobata* and *Sphagneticola calendulacea* to low temperature and weak light characteristic in South China. Sci. Rep..

[25] Song L., Li C., Peng S. (2010). Elevated CO_2_ increases energy-use efficiency of invasive *Wedelia trilobata* over its indigenous congener. Biol. Invasions.

[26] Liu Y., Zhang X., van Kleunen M. (2018). Increases and fluctuations in nutrient availability do not promote dominance of alien plants in synthetic communities of common natives. Funct. Ecol..

[27] Liu Y., Oduor A.M., Zhang Z., Manea A., Tooth I.M., Leishman M.R., Van Kleunen M. (2017). Do invasive alien plants benefit more from global environmental change than native plants?. Global Change Biol..

[28] Liu Y., Liu M., Xu X., Tian Y., Zhang Z., van Kleunen M. (2018). The effects of changes in water and nitrogen availability on alien plant invasion into a stand of a native grassland species. Oecologia.

[29] Parendes L.A., Jones J.A. (2000). Role of light availability and dispersal in exotic plant invasion along roads and streams in the HJ Andrews experimental forest. Oregon. Conserv. Biol..

[30] Lambers H., Oliveira R.S., Lambers H., Oliveira R.S. (2019). Plant water relations. Plant Physiological Ecology.

[31] Lozano Y.M., Aguilar-Trigueros C.A., Flaig I.C., Rillig M.C. (2020). Root trait responses to drought are more heterogeneous than leaf trait responses. Funct. Ecol..

[32] Valladares F., Sanchezgomez D., Zavala M.A. (2006). Quantitative estimation of phenotypic plasticity: Bridging the gap between the evolutionary concept and its ecological applications. J. Ecol..

[33] Gruntman M., Segev U., Tielbrger K. (2020). Shade-induced plasticity in invasive *Impatiens glandulifera* populations. Weed Res..

[34] Ma J. (2013). The Checklist of the Chinese Invasive Plants.

[35] Liu Y., Oduor A.M., Dai Z., Gao F., Li J., Zhang X., Yu F. (2021). Suppression of a plant hormone gibberellin reduces growth of invasive plants more than native plants. Oikos.

[36] Speißer B., Liu Y., van Kleunen M. (2021). Biomass responses of widely and less-widely naturalized alien plants to artificial light at night. J. Ecol..

[37] Valladares F., Wright S.J., Lasso E., Kitajima K., Pearcy R.W. (2000). Plastic phenotypic response to light of 16 congeneric shrubs from a Panamanian rainforest. Ecology.

[38] Pinheiro J., Bates D., DebRoy S., Sarkar D. (2020). Nlme: Linear and Nonlinear Mixed Effects Models.

[39] Aerts R., Chapin F. (1999). The mineral nutrition of wild plants revisited: A re-evaluation of processes and patterns. Advances in Ecological Research.

[40] Koç İ., Nzokou P., Cregg B. (2022). Biomass allocation and nutrient use efficiency in response to water stress: Insight from experimental manipulation of balsam fir, concolor fir and white pine transplants. New Forests.

[41] Kramer-Walter K.R., Laughlin D.C. (2017). Root nutrient concentration and biomass allocation are more plastic than morphological traits in response to nutrient limitation. Plant Soil.

[42] Kleyer M., Trinogga J., Cebrian-Piqueras M.A., Trenkamp A., Flojgaard C., Ejrnaes R., Bouma T.J., Minden V., Maier M., Mantilla-Contreras J. (2019). Trait correlation network analysis identifies biomass allocation traits and stem specific length as hub traits in herbaceous perennial plants. J. Ecol..

[43] Weiner J. (2004). Allocation, plasticity and allometry in plants. Perspect. Plant Ecol. Evol. Syst..

[44] Rehling F., Sandner T.M., Matthies D. (2021). Biomass partitioning in response to intraspecific competition depends on nutrients and species characteristics: A study of 43 plant species. J. Ecol..

[45] Luong J.C., Loik M.E. (2022). Adjustments in physiological and morphological traits suggest drought-induced competitive release of some California plants. Ecol. Evol..

[46] Bloom A.J., Chapin F.S., Mooney H.A. (1985). Resource limitation in plants—An economic analogy. Annu. Rev. Ecol. Syst..

[47] Tsukaya H., Adams W.W., Terashima I. (2018). A consideration of leaf shape evolution in the context of the primary function of the leaf as a photosynthetic organ. The Leaf: A Platform for Performing Photosynthesis.

[48] Quan G., Mao D., Zhang J., Xie J. (2015). Effects of nutrient level on plant growth and biomass allocation of invasive *Chromolaena odorata*. Ecol. Sci..

[49] Martinez K.A., Fridley J.D. (2018). Acclimation of leaf traits in seasonal light environments: Are non-native species more plastic?. J. Ecol..

[50] Bazzaz F.A., Chiarirllo N.R., Coley P.D., Pitelak L.F. (1987). Allocating resources to reproduction and defense. BioScience.

[51] Harper J.L., Ogden J. (1970). The reproductive strategy of higher plants: I. The concept of strategy with special reference to *Senecio Vulgaris*, L.. J. Ecol..

[52] Eziz A., Yan Z., Tian D., Han W., Tang Z., Fang J. (2017). Drought effect on plant biomass allocation: A meta-analysis. Ecol. Evol..

[53] Valliere J.M., Escobedo E.B., Bucciarelli G.M., Sharifi M.R., Rundel P.W. (2019). Invasive annuals respond more negatively to drought than native species. New. Phytol..

[54] Davis M.A., Grime J.P., Thompson K. (2000). Fluctuating resources in plant communities: A general theory of invasibility. J. Ecol..

[55] Goergen E., Daehler C.C. (2001). Reproductive ecology of a native Hawaiian grass (*Heteropogon contortus*; Poaceae) versus its invasive alien competitor (*Pennisetum setaceum*; Poaceae). Int. J. Plant Sci..

[56] Muth N.Z., Pigliucci M. (2007). Implementation of a novel framework for assessing species plasticity in biological invasions: Responses of *Centaurea* and *Crepis* to phosphorus and water availability. J. Ecol..

[57] Sorte C.J., Ibáñez I., Blumenthal D.M., Molinari N.A., Miller L.P., Grosholz E.D., Dukes J.S. (2013). Poised to prosper? A cross-system comparison of climate change effects on native and non-native species performance. Ecol. Lett..

[58] Huang W., Zhang S.B., Liu T. (2018). Moderate photoinhibition of photosystem II significantly affects linear electron flow in the shade-demanding plant *Panax notoginseng*. Front. Plant Sci..

[59] Hussain S., Iqbal N., Ting P., Khan M.N., Liu W., Yang W. (2019). Weak stem under shade reveals the lignin reduction behavior. J. Integr. Agr..

[60] Liu W., Ren M., Liu T., Du Y., Zhou T., Liu X., Liu J., Hussain S., Yang W. (2018). Effect of shade stress on lignin biosynthesis in soybean stems. J. Integr. Agr..

[61] Poorter H., Niinemets U., Ntagkas N., Siebenkas A., Maenpaa M., Matsubara S., Pons T. (2019). A meta-analysis of plant responses to light intensity for 70 traits ranging from molecules to whole plant performance. New. Phytol..

[62] Wright I.J., Reich P.B., Westoby M., Ackerly D.D., Baruch Z., Bongers F., Villar R. (2004). The worldwide leaf economics spectrum. Nature.

[63] Valladares F., Niinemets Ü. (2008). Shade tolerance, a key plant feature of complex nature and consequences. Annu. Rev. Ecol. Evol. Syst..

[64] Liu Y., Dawson W., Prati D., Haeuser E., Feng Y., van Kleunen M. (2016). Does greater specific leaf area plasticity help plants to maintain a high performance when shaded?. Ann. Bot. London.

[65] Wang R., Sun B., Li J., Wang G., Sun J., Wang X., Zhong R. (2012). Effects of light intensity on the phenotypic plasticity of invasive species *Ambrosia trifida*. J. Appl. Ecol..

