# Supplementary material for: Differences in Phenotypic Plasticity between Invasive and Native Plants Responding to Three Environmental Factors
Source: Life (Basel). 2022 Nov 25;12(12):1970. doi: 10.3390/life12121970 (PMC9781723; doi:10.3390/life12121970)
Supplement: Supplementary file 1 [file life-12-01970-s001.zip › life-1999436-supplementary.pdf]

Table S1 Mean (± SE) values of height final, specific leaf area (SLA), leaf number, total biomass, root mass fraction (RMF) and leaf mass fraction (LMF) at the end of experiment of native and invasive species growing under nutrient, light and water treatments.

Variables	Treat	Asteraceae		Amaranthaceae		Araliaceae	
		Invasive	Native	Invasive	Native	Invasive	Native
Height Final	Low nutrient	125.3 ± 8.13	108.9 ± 5.37	86.2 ± 7.27	43.5 ± 9.26	91.2 ± 7.52	41.8 ± 7.28
	High nutrient	126.3 ± 7.84	128.7 ± 4.96	78.2 ± 9.36	100.1 ± 12.36	170.6 ± 17.71	69.3 ± 6.41
	Low water	73.5 ± 3.49	76.3 ± 5.07	52.7 ± 2.42	45.5 ± 6.20	89.6 ± 10.02	56.1 ± 2.25
	High water	89.9 ± 4.09	93.7 ± 6.83	64.5 ± 5.43	61.3 ± 8.00	92.3 ± 8.88	72.2 ± 5.48
	Shading	57.5 ± 2.22	60.2 ± 1.92	70.3 ± 6.33	42.3 ± 8.26	97.3 ± 10.70	46.9 ± 6.80
	Ambient light	119.9 ± 15.57	132.7 ± 6.92	134.3 ± 5.21	99.7 ± 9.73	109.2 ± 8.66	68.8 ± 8.21
Leaf Number	Low nutrient	238.1 ± 23.73	190.6 ± 18.37	223.6 ± 34.68	206.0 ± 50.81	43.4 ± 5.99	16.3 ± 5.57
	High nutrient	431.0 ± 64.21	377.5 ± 49.35	240.3 ± 35.62	349.9 ± 76.94	75.6 ± 7.54	139.8 ± 32.51
	Low water	146.8 ± 10.22	137.3 ± 6.89	161.9 ± 17.47	101.6 ± 12.55	26.0 ± 2.11	86.1 ± 8.95
	High water	227.6 ± 12.67	195.8 ± 14.34	219.4 ± 26.34	180.3 ± 16.92	42.3 ± 3.16	157.3 ± 17.97
	Shading	34.9 ± 4.19	42.4 ± 3.27	76.6 ± 19.31	96.1 ± 23.95	25.8 ± 9.04	12.8 ± 3.40
	Ambient light	325.8 ± 31.00	360.6 ± 40.82	719.9 ± 49.72	530.5 ± 91.34	64.3 ± 7.17	56.9 ± 19.38
SLA	Low nutrient	410.8 ± 21.24	394.7 ± 29.45	420.2 ± 19.46	619.3 ± 48.15	277.2 ± 11.99	511.0 ± 21.74
	High nutrient	413.2 ± 29.99	424.2 ± 13.76	448.5 ± 16.66	412.9 ± 42.31	255.7 ± 11.88	533.3 ± 20.58
	Low water	298.8 ± 12.26	341.3 ± 17.98	257.5 ± 8.30	343.1 ± 23.29	224.3 ± 7.59	378.5 ± 14.88
	High water	358.9 ± 13.63	391.7 ± 21.03	286.3 ± 7.89	333.7 ± 25.35	235.5 ± 9.71	402.3 ± 11.87
	Shading	633.2 ± 33.68	612.3 ± 19.76	602.5 ± 30.68	680.2 ± 50.18	408.7 ± 7.05	1058.6 ± 310.56
	Ambient light	428.8 ± 15.56	475.7 ± 20.47	388.8 ± 17.37	460.8 ± 36.75	266.3 ± 7.98	520.8 ± 23.64
Total Biomass	Low nutrient	5.6 ± 0.90	3.9 ± 0.41	4.8 ± 0.93	3.7 ± 1.30	3.7 ± 0.59	0.2 ± 0.05
	High nutrient	10.2 ± 1.38	8.6 ± 0.83	5.1 ± 1.04	9.4 ± 2.67	8.8 ± 0.98	1.2 ± 0.36
	Low water	3.9 ± 0.27	3.9 ± 0.33	4.4 ± 0.20	2.8 ± 0.39	3.1 ± 0.41	1.6 ± 0.23
	High water	5.9 ± 0.19	5.0 ± 0.29	6.3 ± 0.19	4.6 ± 0.62	3.8 ± 0.37	2.5 ± 0.33
	Shading	0.6 ± 0.05	0.7 ± 0.06	0.9 ± 0.31	1.2 ± 0.29	1.3 ± 0.33	0.1 ± 0.03
	Ambient light	8.5 ± 0.63	7.9 ± 1.04	16.9 ± 1.22	14.2 ± 2.53	7.3 ± 0.97	0.5 ± 0.18
RMF	Low nutrient	11.2 ± 1.29	9.8 ± 1.02	13.6 ± 1.65	10.5 ± 1.06	5.6 ± 0.47	10.1 ± 1.37
	High nutrient	5.7 ± 0.59	6.7 ± 0.73	7.1 ± 0.59	7.0 ± 1.07	2.9 ± 0.23	4.9 ± 1.31
	Low water	14.3 ± 0.82	15.1 ± 1.14	22.6 ± 1.18	11.8 ± 1.27	9.2 ± 0.63	11.2 ± 1.52
	High water	12.5 ± 0.83	12.9 ± 0.69	22.7 ± 0.51	14.6 ± 1.57	7.7 ± 0.32	9.3 ± 0.72
	Shading	5.7 ± 0.70	6.4 ± 0.75	5.9 ± 1.21	5.9 ± 1.05	2.6 ± 0.25	7.4 ± 2.31
	Ambient light	6.6 ± 0.74	5.4 ± 0.23	8.6 ± 0.53	9.6 ± 1.01	3.6 ± 0.55	5.8 ± 1.20
LMF	Low nutrient	33.2 ± 1.91	36.5 ± 0.93	29.6 ± 2.01	36.4 ± 2.69	56.9 ± 2.42	30.0 ± 4.81
	High nutrient	39.4 ± 3.01	36.7 ± 0.79	36.0 ± 1.38	36.6 ± 1.63	67.7 ± 1.59	43.9 ± 3.54
	Low water	29.8 ± 0.83	28.7 ± 2.74	20.3 ± 1.64	34.9 ± 4.43	38.9 ± 1.37	36.5 ± 3.36
	High water	29.5 ± 0.59	30.5 ± 1.26	22.1 ± 0.68	29.3 ± 2.49	44.8 ± 2.52	36.9 ± 1.55
	Shading	35.4 ± 1.02	38.2 ± 0.89	34.9 ± 1.86	40.3 ± 2.98	68.6 ± 4.73	32.4 ± 5.61
	Ambient light	34.9 ± 0.63	37.7 ± 0.69	33.1 ± 0.42	30.8 ± 1.38	60.3 ± 2.17	44.2 ± 3.61

Table S2 Phenotypic plasticity index of height final, specific leaf area (SLA), leaf number, total biomass, root mass fraction (RMF) and leaf mass fraction (LMF) of six species under nutrient, light and water treatments.

Variables	Treat	Asteraceae		Amaranthaceae		Araliaceae	
		Invasive	Native	Invasive	Native	Invasive	Native
Height Final	Nutrient	0.008	0.154	0.093	0.566	0.465	0.397
	Water	0.182	0.186	0.184	0.258	0.030	0.223
	Light	0.521	0.546	0.477	0.576	0.109	0.317
Leaf Number	Nutrient	0.448	0.495	0.069	0.411	0.426	0.884
	Water	0.355	0.299	0.262	0.436	0.385	0.452
	Light	0.893	0.882	0.894	0.819	0.599	0.776
SLA	Nutrient	0.006	0.070	0.063	0.333	0.078	0.042
	Water	0.168	0.129	0.101	0.027	0.048	0.059
	Light	0.323	0.223	0.355	0.323	0.348	0.508
Total Biomass	Nutrient	0.450	0.549	0.060	0.601	0.582	0.872
	Water	0.321	0.203	0.299	0.384	0.171	0.348
	Light	0.935	0.913	0.942	0.918	0.822	0.796
RMF	Nutrient	0.491	0.317	0.477	0.328	0.487	0.516
	Water	0.127	0.151	0.003	0.189	0.164	0.175
	Light	0.128	0.160	0.312	0.384	0.259	0.213
LMF	Nutrient	0.158	0.004	0.179	0.006	0.159	0.317
	Water	0.010	0.058	0.081	0.161	0.130	0.011
	Light	0.012	0.011	0.050	0.236	0.120	0.268
